# Health-related quality of life among older adults who experienced the Pohang earthquake in South Korea: A cross-sectional survey

**DOI:** 10.1186/s12955-022-01944-8

**Published:** 2022-03-04

**Authors:** Eun-Mi Kim, Gwang Suk Kim, Heejung Kim, Chang Gi Park, Ogcheol Lee, Betty Pfefferbaum

**Affiliations:** 1 College of Nursing, Research Institute of Nursing Science, Pusan National University, Yangsan, Gyeongsangnam-do Republic of Korea; 2 Present Address: Mo-Im Kim Nursing Research Institute, College of Nursing, Yonsei University, Seoul, Republic of Korea; 3 College of Nursing, University of Illinois, Chicago, IL USA; 4 Red Cross College of Nursing, Chung-Ang University, Seoul, Republic of Korea; 5 Department of Psychiatry and Behavioral Sciences, College of Medicine, University of Oklahoma Health Sciences Center, Oklahoma City, USA

**Keywords:** Older adults, Quality of life, Depression, Social support, Community integration, Earthquakes, Disasters

## Abstract

**Background:**

Earthquakes are global natural disasters and can cause loss of property, livelihood and affect human health. A 5.4 magnitude earthquake, the Pohang earthquake, occurred in South Korea in 2017. In this study, based on a health-related quality of life (HRQOL) conceptual model, we examined the HRQOL and its associated factors among older adults who had experienced the earthquake.

**Methods:**

A cross-sectional study was conducted with a quota sample of 312 older adults living in eight villages of a district that was the most damaged area during the Pohang earthquake. Data were collected from January 15–March 19, 2019, via face-to-face interviews using structured questionnaires. Structural equation modeling was performed to explore the associations among depression, posttraumatic stress symptoms, community resilience, social support, disaster preparedness, and HRQOL.

**Results:**

The mean age of the participants was 77.93 ± 6.11 years. HRQOL scores were 49.85 ± 18.07 (physical health), 50.16 ± 18.75 (psychological health), 61.93 ± 19.20 (social relations), and 49.53 ± 16.37 (environment). The structural equation modeling analysis showed a good fit. Depression had direct (*β* =  − 2.21; *p* < 0.001), indirect (*β* =− 0.23; *p* < 0.001), and total effects on HRQOL (*β* =  − 2.44; *p* < 0.001). Community resilience (*β* = 6.05; *p* = 0.001) and social support (*β* = 0.12, *p* = 0.001) had direct and total effects on HRQOL. Disaster preparedness had indirect (*β* = 0.40; *p* = 0.001) and total (*β* = 0.69, *p* = 0.031) effects on HRQOL. In contrast, posttraumatic stress symptoms did not have significant effects on HRQOL.

**Conclusions:**

Our findings indicated that lower depression, higher community resilience, social support, and disaster preparedness were associated with increased HRQOL. Thus, it is helpful to decrease depression and strengthen community resilience, social support, and disaster preparedness to promote HRQOL among older adults who have experienced earthquakes. These results can inform the development of HRQOL in socio-psychological improvement programs for older adults in community health centers and disaster-relief psychological support centers.

## Background

Worldwide, in 2019, 396 natural disasters causing 11,755 deaths, injuring 95 million people, and resulting in $103 billion U.S. in economic losses were recorded by the Emergency Events Database, maintained by Center for Research on the Epidemiology of Disasters [1]. Asia was one of the most highly impacted regions and experienced a burden that accounted for 40% of disasters, 45% of deaths, and 74% of the total number of people affected [1]. On November 15, 2017, a 5.4 magnitude earthquake occurred in Pohang city, South Korea. The earthquake caused extensive damage: 544 households were damaged, 1,392 people were displaced, and, reportedly, financial losses amounted to 55,057 million Korean won (approximately USD 48 million) [2].

Earthquakes lead to undesirable changes in lifestyle and harm residents’ physical and psychological health [3–5], which ultimately may lead to decreased health-related quality of life (HRQOL) [4, 6, 7]. Older adults are more vulnerable in disaster situations as they are prone to experience greater issues owing to less mobility, physical disability, and impaired cognitive abilities as compared to their younger counterparts [8, 9]. A previous study reported that the HRQOL scores of older adults who experienced earthquakes are lower than those of other adults who have not experienced earthquakes [10]. A systematic review reported that older adults are more likely to have a higher mortality rate during earthquakes than other demographic groups [6]. Therefore, in the event of a disaster, careful attention should be paid to older adults.

Studies such as by the Bam [8], Chi-Chi [11], L’Aquila [10, 12], Niigata-chuetsu [13], and Sichuan [4] earthquakes identified older adults’ HRQOL in various regions and post-earthquakes. These studies also investigated various factors associated with older adults’ HRQOL, which included older age [4, 8, 10], sex [8, 10, 13], education level [4, 10], physical disease [4, 13], injury [8], difficulty with activities of daily living (ADL), depression [9], post-disaster stress, living alone, poor living conditions [8], and loss of family members [4].

Social and environmental factors are also associated with HRQOL, which indicates the importance of community efforts toward recovery and social support. Social isolation [12], disruption of social networks [12], and reduced social support [9] are associated with decreased HRQOL. Furthermore, older adults have difficulties in acquiring disaster information during earthquakes and maintaining social networks [12]. In South Korea, older adults often live in an area for a long time and have close relationships with their neighbors. In the event of a disaster, if social support decreases and social isolation occurs, older adults may become more vulnerable [9, 11]. Additionally, if many patients are admitted in hospitals, older people may have difficulties accessing the medical services they need [8].

After the Pohang earthquake, social interest in disaster management and community resilience—a comprehensive concept related to disaster risk reduction activities and community effort for recovery—increased in South Korea [2, 14, 15]. In disaster situations, essential human needs like food, clothing, and shelter are threatened; thus, the government’s disaster management organization is important, including disaster policy, resource distribution, and relief for victims [16]. Satisfaction with economic and social rights is positively associated with HRQOL [16, 17]. Community efforts to recover from a disaster, including rescue policies [18], economic and social rights [16], community resilience [19, 20], and financial support [20] are also associated with HRQOL. Disaster preparedness, which includes disaster risk reduction activities and health-protective behaviors during life-threatening disasters is also positively associated with HRQOL [19]. Studies have also emphasized the importance of social support [12, 21].

A majority of extant studies focus on victims’ basic rights and HRQOL-related policies. Few studies have identified the association between social environmental factors such as community resilience, disaster preparedness, social support, and HRQOL after an earthquake. Community resilience and social support are expected to improve victims’ HRQOL by alleviating individual psychological difficulties [15, 22]. However, owing to the lack of empirical examination in disaster practice among vulnerable older adults, it is difficult to grasp the major effects and mediated effects of community resilience, disaster preparedness, and social support on HRQOL.

Several studies have investigated the factors associated with older adults’ HRQOL in diverse overseas settings after earthquakes have occurred; however, few studies have focused on vulnerable older adults in South Korea. Additionally, researchers have identified the associations between depression, posttraumatic stress symptoms (PTSS), community resilience, disaster preparedness, and social support and HRQOL among adults [16, 18, 19]; however, the multidimensional factors, including individual and social environmental aspects among older adults, require elucidation. In areas where earthquakes have occurred, conducting surveys regarding vulnerable populations’ HRQOL may provide specific insights and valuable information to improve healthcare services, disaster preparedness, psychological supporting programs during and after disasters, and disaster policies and management.

### Conceptual framework

The conceptual framework of this study is based on the modified HRQOL model developed by Wilson and Cleary [23] and revised by Zubritsky et al. [24]. HRQOL includes aspects such as functional, physical, and emotional health [25]. The HRQOL model developed by Wilson and Cleary [23] describes the multidimensional domains of health and comprehensively specifies a series of critical concepts by using a causal pathway. Zubritsky et al. [24] revised this model by focusing on high-priority performances of older adults such as cognition and behavior. The modified HRQOL model expanded the existing conceptual framework by establishing a bi-directional causal relationship of the multidimensional domains.

### Aim and objectives

The current study aimed to identify associations between HRQOL and multidimensional factors among older adults in areas where the earthquake had occurred. The specific objectives were as follows: 1) to identify the status of older adults’ HRQOL; 2) to establish a hypothetical model that describes the associations among community resilience, social support, disaster preparedness, depression, PTSS, ADL, instrumental ADL (IADL), and HRQOL based on the above-mentioned theoretical model; and 3) to evaluate the suitability of the hypothetical model using structural equation modeling (SEM).

## Methods

### Design and setting

The current study was conducted using a cross-sectional design. It was conducted in eight villages in Heunghae district, Pohang city, South Korea—one of the areas most damaged by the earthquake.

### Participants

**Fig. 1:**
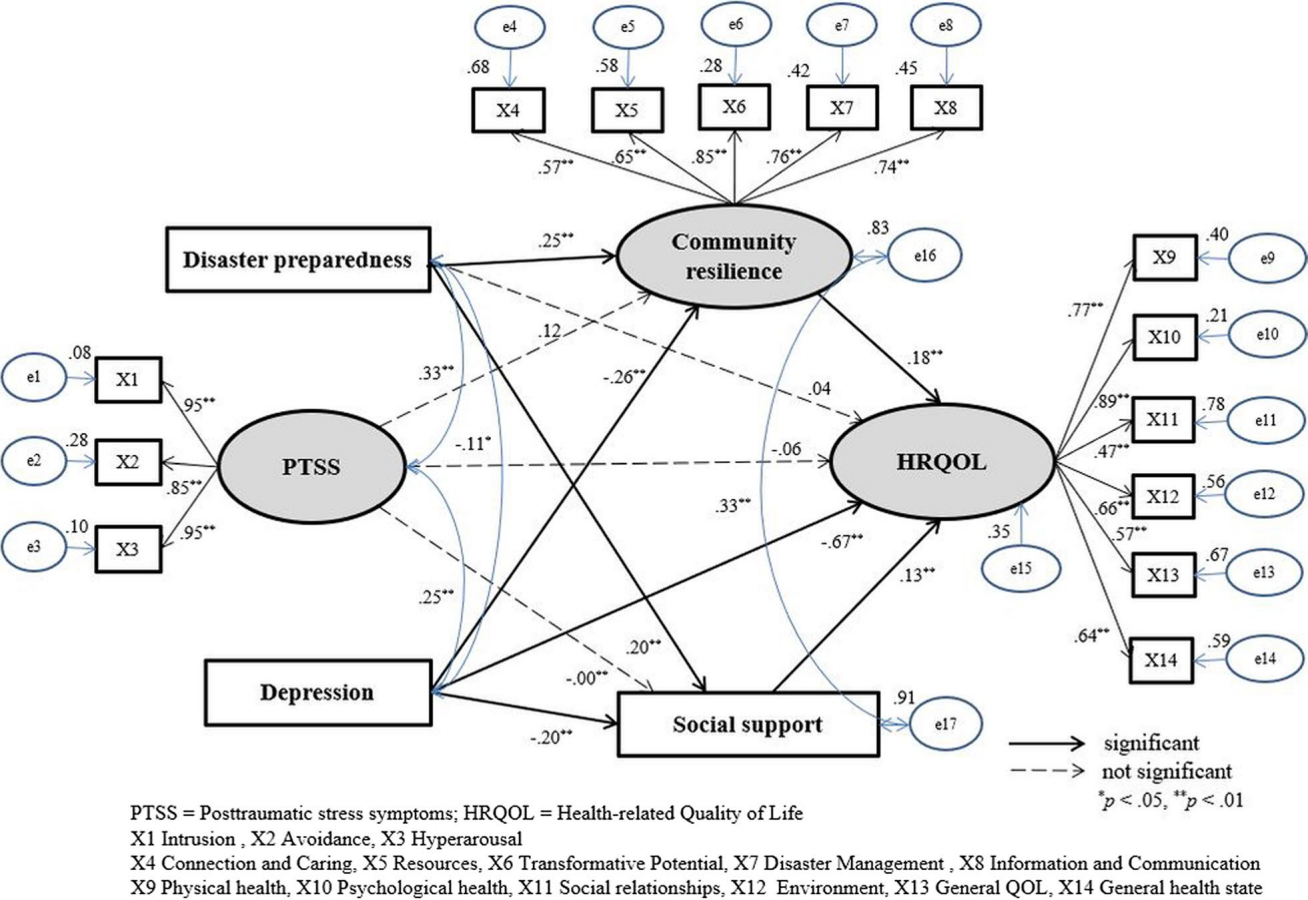
Structural equation model of modified model and standardized path coefficients

Participants were selected by proportional quota sampling. The number of older adults were calculated proportionately based on the actual number of older adults aged 65 or older living in each of the eight villages compiled by the Heunghae district Administrative Welfare Center (June 27, 2018). The study sample comprised 500 older adults from the eight villages, who were recruited according to the proportions as follows: Namsung-ri (26.0%), Masan-ri (19.9%), Mangchen-ri (4.8%), Sungnae-ri (2.8%), Yaksung-ri (10.7%), Oksung-ri (21.7%), Jungsung-ri (10.5%), and Haksung-ri (3.6%). Participant inclusion criteria were as follows: (a) older adults aged ≥ 65 years; (b) scoring more than 24 points on the Korean version of the Mini-Mental State Examination; and (c) able to communicate, understand, and respond to questionnaires in Korean. Older adults who had difficulty understanding and responding to the questionnaire (e.g., owing to cognitive disabilities) were excluded. A total of 332 older adults participated in this study during the data collection period. After excluding 20 insufficient responses, 312 responses from older adults were analyzed (total response rate = 62.4%). This study used a priori sample size calculator for SEM; the minimum sample size to detect a small effect size, at a desired power of 0.80 with 17 latent variables and three observed variables, was calculated as 296 (Fig. 1) [26]. Therefore, the 312 participants in the current study met the minimum sample size requirements.

### Ethical considerations

This study obtained ethical approval from the appropriate Institutional Research Board (no. Y-2018–0111). Participants were provided with explanations about the research, such as purpose, necessity, and data collection method. Participants voluntarily agreed to participate and provided written consent. In order to ensure anonymity and confidentiality, anonymized data was collected. Those who participated were provided with a transportation fee reimbursement. Questionnaires and databases were confidentially managed.

### Instruments

#### Health-related quality of life

HRQOL was assessed using the abbreviated brief version of the World Health Organization (WHO) questionnaire (WHOQOL-BREF), which consists of 26 items, and is a simplified version of the 100-item World Health Organization Quality of Life scale. The WHOQOL-BREF includes physical health, psychological health, social relations, and environmental domains as well as two individual items about subjective quality of life and health conditions. We used the Korean version of the WHOQOL-BREF, which was adapted by Min and colleagues [27]. Responses were measured with a five-point Likert scale. Higher scores indicated better quality of life [28]. The scale has good reliability: Cronbach’s alphas were 0.90, and 0.89 [27] among the South Korean population. In this study, Cronbach’s alpha was 0.86.

#### Community resilience

Community resilience was assessed using the Communities Advancing Resilience Toolkit (CART) Assessment Survey developed by Pfefferbaum and colleagues [29, 30]. In this study, the Korean version of CART was developed and utilized in compliance with the WHO’s translation guidelines [31]. Further, it was assessed for its content validity, construct validity, and reliability. All values for validity and reliability were found to be acceptable. The CART includes 26 items with five core interrelated subdomains: (a) connection and caring, (b) resources, (c) transformative potential, (d) disaster management, and (e) information and communication. Responses were measured with a five-point Likert scale (from 1 = “strongly disagree” to 5 = “strongly agree”). The Cronbach’s alphas were 0.87 previously [29] and 0.91 in this study.

#### Social support

Social support was assessed using the Crisis Support Scale developed by Joseph and colleagues [32]. In this study, the Korean version was developed and utilized in compliance with WHO’s translation guidelines [31]. After a disaster, the degree of social support might vary over time. The measure contains seven items, each asked on two separate occasions: within three months following the event (Time 1) and within the last three months (Time 2). In this study, to identify the overall degree of social support, the average values for Time 1 and Time 2 were calculated. Questions concerned (a) the availability of others, (b) contact with other survivors, (c) confiding in others, (d) emotional support, (e) practical support, and (f) satisfaction with support. Responses were measured with a seven-point Likert scale (from 1 = “never” to 7 = “always”). Higher scores indicate greater support. A total Time 1 crisis support score was obtained by summating items 1, 3, 5, 7, 9, and 11 (reversed). Similarly, a total Time 2 crisis support score was obtained by summating items 2, 4, 6, 8, 10, and 12 (reversed). The Cronbach’s alphas were 0.80 previously [32] and 0.86 in this study.

#### Disaster preparedness

Disaster preparedness refers to the extent of a community’s or an individual’s preparedness to reduce earthquake damages, which includes disaster risk reduction activities and disaster preventive actions. We developed 13 items about disaster preparedness based on literature [15] and the National Action Guidelines for Earthquakes, developed by the South Korean government [33]. Items were validated using content validity from a panel of experts in disaster practice and reseach. Each item had two response options: “yes” or “no.” The scores of each question were added: one point for “yes” and zero points for “no.” Cohen’s Kappa was 0.46 in this study.

#### Depression

Depression was assessed using the short form of the Geriatric Depression Scale-15 (GDS-15), which was developed based on the standardized GDS by Yesavage and colleagues [34]. Kee [35] developed the Korean version of the GDS-15 and verified its validity and reliability. The scale consists of 15 items. Each item has two response options: “yes” or “no.” Total scores range from 0–15 and higher scores indicate more severe depression. Cronbach’s alphas were 0.94 [34] and 0.88 [35] previously. In this study, the Kuder–Richardson Formula 20 was 0.86. Statistically, in this case, the KR-20 is a more appropriate method than Cronbach's alpha for testing reliability, as this scale consisted of dichotomized response pattern, and the value was 0.86, which indicates high internal consistency.

#### Posttraumatic stress symptoms

PTSS were assessed using the Korean version [36] of the 22-item Impact of Event Scale-Revised (IES-R) [37], which assesses posttraumatic stress disorder symptoms after a specific traumatic stressor. It is one of the most widely used measures in trauma-related research. It consists of three subscales corresponding to the three dimensions of the criteria for posttraumatic stress disorder outlined in the fourth edition of the Diagnostic and Statistical Manual of Mental Disorders [38]: intrusion (eight items), avoidance (eight items), and hyperarousal symptoms (six items). Responses were measured using a five-point Likert scale (0 = “not at all” to 4 = “often”). Cronbach’s alphas were 0.79 previously [37] and 0.95 in this study.

#### Activities of daily living

Activities of daily living were assessed with the Korean version of the ADL scale developed by Won and colleagues [39]. The scale has 17 items and includes two parts: ADL and IADL. The ADL component has seven items related to dressing, washing one’s face, bathing, eating, moving, going to the bathroom, and continence. The IADL has ten items related to participants’ abilities concerning grooming, housekeeping, preparing meals, laundry, transportation use, money management, telephone use, shopping, going out to nearby places, and taking one’s medicine. The total score is calculated as the sum of each item using a three-point Likert scale. The ADL scores range from 7 to 21, while the IADL scores range from 10 to 30. Higher scores indicate better physical health. Cronbach’s alphas were 0.94 previously [39], and 0.78 in this study.

#### Socio-demographic factors

The socio-demographic factors that we measured included age, sex, marital status, education level, religion, family cohabitation, working conditions, and monthly income.

The tools, such as, WHOQOL-BREF, GDS-15, IES-R, and ADL, were used in this study and have already been tested for reliability in the Korean population. The 312 older adults were used to evaluate reliability for each variable and to see if the results reflect whether the tools well measured the concept for study subjects in this study. The following tools were used for the first time in a Korean sample (CART, Crisis support scale, and Disaster preparedness), with the study used to confirm the reliability of the subjects in this study to provide evidence on the reliability for future research.

### Data collection

Data were collected from January 15 to March 19, 2019. Surveyors visited 22 local facilities, such as centers for senior citizens, schools for senior citizens, and temporary residential shelters in eight villages in Heunghae district to collect data. Ten surveyors conducted face-to-face interviews using structured questionnaires. Prior to data collection, educational training was conducted for the ten surveyors regarding the survey purpose, inclusion/exclusion criteria, data collection methods, obtaining consent, among other things. Data were collected after obtaining permission and cooperation from 22 local facilities. Research information was posted at these local facilities to recruit participants in advance. It took each participant about 30–40 min to complete the questionnaire.

### Data analysis

Data analysis was performed using IBM SPSS statistics version 23.0 (IBM Corp., Armonk, NY, USA) and Stata version 13.0 (Stata Corp., College Station, TX, USA). Descriptive statistics were calculated for socio-demographic characteristics and study variables, including percentage, frequency, mean, and standard deviation. T-tests, one-way analyses of variance, and Pearson’s correlation coefficients were used to identify the differences and relationships between study variables. SEM was used to test the model fit between the hypothetical model and the collected data. Multicollinearity was checked with tolerance and variation inflation factors. The multivariate normality of the sample was identified using Doornik–Hansen test [40]. Although it is recommended that the multivariate normality assumption is met for SEM analysis, this is rare among such data. If univariate normality is satisfied, the data are considered appropriate for SEM analysis [41]. Univariate normality is not satisfied if the skewness index > 3 and the kurtosis index > 7. The data from the current study did not satisfy these multivariate normality assumptions. Except for ADL and IADL, the univariate normality assumptions were satisfied. The ADL and IADL were initially included in the hypothetical model as exogenous variables, but they were excluded in the results of the final analysis. Bootstrapping was used to verify the significance of the direct, indirect, and total effects of the hypothetical model. The evaluation of the goodness of model fit is presented in Table 3 and based on the following criteria: χ^2^ (CMIN), normed χ^2^ (CMIN/df) ≤ 3, standard root mean residual (SRMR) ≤ 0.05, 0.06 ≤ root mean squared of approximation (RMSEA) ≤ 0.08, comparative fit index (CFI) ≥ 0.90, and Tucker–Lewis index (TLI) ≥ 0.90.

## Results

### General characteristics of the participants

Participants’ general characteristics are presented in Table 1. Of the 312 older adults, the mean age of participants was 77.93 ± 6.11 years: 160 participants (51.3%) were of ages 75–84 years and 104 (33.3%), 65–74 years. Most were women (n = 217; 69.6%), 166 (53.2%) were married, 166 (53.2%) had only an elementary school level of education, 182 (58.3%) were older adults living with their families, and 234 (75.0%) were unemployed.

**Table 1:** General characteristics of participants (*N* = 312)

Characteristics	Categories	*n* (%)	Mean ± *SD*
Age (year)			77.93 ± 6.11
	65–74	104 (33.3)	
	75–84	160 (51.3)	
	≥ 85	48 (15.4)	
Sex	Male	95 (30.4)	
	Female	217 (69.6)	
Marriage	Married	166 (53.2)	
	Bereavement or divorce	146 (46.8)	
Education	No school	58 (18.6)	
	Elementary school	166 (53.2)	
	Middle school	59 (18.9)	
	≥ High school	29 (9.3)	
Religion	None	119 (38.1)	
	Buddhism	115 (36.9)	
	Christianity	65 (20.8)	
	Others	13 (4.2)	
Family cohabitation	Living alone	130 (41.7)	
	Living with family	182 (58.3)	
Working condition	Full time	31 (9.9)	
	Part time	47 (15.1)	
	Without job	234 (75.0)	
Monthly income (n = 303; 10,000 won)	< 50	160 (52.8)	
	50–99	94 (31.0)	
	≥ 100	49 (16.2)	

SD = Standard deviation

### Descriptive statistics of the study variables

The mean overall HRQOL score among older adults was 51.39 ± 14.60. The mean community resilience score was 3.24 ± 0.64 and the mean PTSS score was 1.90 ± 0.97. The mean depression score was 6.57 ± 4.23, disaster preparedness was 7.33 ± 2.30, and social support was 28.30 ± 7.06 (Table 2).

**Table 2:** Descriptive statistics of observed variables (*N* = 312)

Variable	Range	Mean ± *SD*	Min	Max
HR-QOL	0–100	51.39 ± 14.60	11.71	91.87
1. Physical health	0–100	49.85 ± 18.07	0.0	96.43
2. Psychological health	0–100	50.16 ± 18.75	4.17	100.00
3. Social relationships	0–100	61.93 ± 19.20	0	100.00
4. Environment	0–100	49.53 ± 16.37	3.13	93.75
5. General quality of life	0–100	53.44 ± 20.00	0	100.00
6. General health state	0–100	43.42 ± 26.91	0	100.00
Community resilience	1–5	3.24 ± .64	1.23	5.00
1. Connection and caring	1–5	3.69 ± .71	1.00	5.00
2. Resources	1–5	2.75 ± .85	1.00	5.00
3. Transformative potential	1–5	3.12 ± .75	1.13	5.00
4. Disaster management	1–5	3.30 ± .95	1.00	5.00
5. Information and communication	1–5	3.47 ± .90	1.00	5.00
Social support	7–42	28.30 ± 7.06	11.00	42.00
1. Time 1; in the 3 months after events	7–42	29.41 ± 7.42	11.00	42.00
2. Time 2; last 3 months	7–42	27.19 ± 7.52	8.00	42.00
Disaster preparedness	0–13	7.33 ± 2.30	0	13.00
Depression	0–15	6.57 ± 4.23	0	15.00
PTSS	0–4	1.90 ± .97	0	4.00
1. Intrusion	0–4	1.95 ± .98	0	4.00
2. Avoidance	0–4	1.85 ± 1.00	0	4.00
3. Hyperarousal	0–4	1.92 ± 1.12	0	4.00
ADL	7–21	7.07 ± .32	7.00	9.00
IADL	10–30	10.38 ± 1.21	10.00	19.00

SD = Standard deviation; HR-QOL = Health-related quality of life; PTSS = posttraumatic stress symptoms; ADL = activities of daily living; IADL = instrumental activities of daily living

### Structural model of HRQOL

The hypothetical model based on the modified HRQOL model developed by Wilson and Cleary [23] and revised by Zubritsky et al. [24] from the SEM analysis is illustrated in Fig. 1. The SEM model fit indices are presented in Table 3. The model fit of the hypothetical model was adequate: χ^2^ = 292.61 (*p* < 0.001), normed χ^2^ = 2.73, SRMR = 0.05, RMSEA = 0.07, CFI = 0.93, and TLI = 0.91. The hypothetical model was modified considering modification indices and theoretical background by connecting the error terms with the highest modification indices. In the modified model of this study, error terms with e16 and e17 were connected. The fit of the modified model was more adequate than that of the hypothetical model: χ^2^ = 253.55 (*p* < 0.001), normed χ^2^ = 2.39, SRMR = 0.04, RMSEA = 0.06, CFI = 0.94, and TLI = 0.93. Therefore, the results showed that the goodness-of-fit of the modified model was acceptable.

**Table 3:** Structural equation models and model fit indices (*N* = 312)

Model fit index	Acceptable criteria	Hypothetical model	Modified model
χ^2^ (CMIN)	Lower	292.61	253.55
Normed χ2 (CMIN/*df*)	≤ 3.0	2.73	2.39
*p*	> 0.05	< 0.001	< 0.001
SRMR	≤ 0.05	0.05	0.04
RMSEA	≤ 0.05 (good)	0.07	0.06
	≤ 0.08 (acceptable)		
CFI	≥ 0.90	0.93	0.94
TLI	≥ 0.90	0.91	0.93
Parsimonious fit index			
AIC	Closer to “0”	30,016.47	2858.56

SRMR = Standardized root mean squared residual; RMSEA = Root mean square error of approximation; CFI = Comparative fit index; TLI = Tucker-Lewis Index; AIC = Akaike information criterion

Standardized regression coefficients (*β*) for each path in the modified model, along with *p*-values, are presented in Fig. 1. Depression (*β* = − 0.67, *p* < 0.01) had significant negative effects on participants’ HRQOL. Community resilience (*β* = 0.18, *p* < 0.01) and social support (*β* = 0.13, *p* < 0.01) had significant positive effects on participants’ HRQOL.

To identify the direct, indirect, and total effects on HRQOL, the results of effect analysis using bootstrapping are presented in Table 4. Depression had significant negative direct (*β* =  − 2.21; *p* < 0.001), indirect (*β* = − 0.23; *p* < 0.001), and total (*β* =  − 2.44; *p* < 0.001) effects on HRQOL. Community resilience (*β* = 6.05; *p* = 0.001) and social support (*β* = 0.12, *p* = 0.001) had significant direct and total effects on HRQOL. Disaster preparedness had significant indirect (*β* = 0.40; *p* = 0.001) and total (*β* = 0.69, *p* = 0.031) effects on HRQOL. However, PTSS did not have significant effects on HRQOL.

**Table 4:** Standardized direct, indirect, and total effects of the modified model (*N* = 312)

Endogenous variable	Exogenous variable	Total effect		Direct effect		Indirect effect	
		*β*	*p*	*β*	*p*	*β*	*p*
HRQOL	← Depression	− 2.44	< 0.001	− 2.21	< 0.001	− 0.23	< 0.001
	← Community resilience	6.05	0.001	6.05	0.001	–	–
	← Social support	0.12	0.008	0.12	0.008	–	–
	← PTSS	− 0.07	0.403	− 0.11	0.193	0.03	0.164
	← Disaster preparedness	0.69	0.031	0.28	0.359	0.40	< 0.001
Community resilience	← Depression	− 0.02	< 0.001	− 0.02	< 0.001	–	–
	← Disaster preparedness	0.04	< 0.001	0.04	< 0.001	–	–
	← PTSS	0.00	0.060	0.00	0.060	–	–
Social support	← Depression	− 0.67	< 0.001	− 0.67	< 0.001	–	–
	← Disaster preparedness	1.17	0.002	1.17	0.002	–	–
	← PTSS	− 0.00	0.954	− 0.00	0.954	–	–

Bootstrap replications 50; HRQOL = Health-related Quality of Life; PTSS = Posttraumatic stress symptoms

## Discussion

Disasters such as earthquakes could do extensive damage to the general health of older adults and reduce their HRQOL. To the best of our knowledge, this study was the first attempt to identify the degree of HRQOL and the paths between associated factors and HRQOL among older adults who experienced the Pohang earthquake in South Korea. Because earthquakes are relatively rare in Korea, support systems for vulnerable populations are insufficient. Recently, advocating for the health of vulnerable older adults in areas where disasters have occurred has become a noteworthy phenomenon in South Korea. Thus, we constructed a hypothetical model based on the HRQOL model [23, 24]. This study verified the suitability of the model and the significance of its paths and direct, indirect, and total effects of factors on HRQOL among older adults.

The HRQOL scores among the participating older adults were 49.88 for physical health, 49.98 for psychological health, 61.69 for social relations, and 49.52 for environment. These results were similar to the findings of Xie and colleagues [9], who used the same measuring tool for older adults (mean age = 74.4 years) living in temporary facilities after the 8.0 magnitude Wenchuan earthquake in China (56.2 for physical health, 45.7 for psychological health, 64.2 for social relations, and 52.9 for the environment). Further, in a post-earthquake follow-up study [10], the HRQOL perceived by adults and older individuals who experienced the L’Aquila earthquake in Italy was somewhat higher than that reported in this study.

Xie and colleagues [9] assert that social attention should be paid to the HRQOL of vulnerable older adults in chaotic disaster situations because they are susceptible to lower HRQOL than younger adults. The current study was conducted based on the assumption that older adults might be more prone to chronic diseases, have limited physical abilities, have difficulty accessing disaster information, and experience a lack of appropriate health services [8, 9]. Thus, community leaders and healthcare providers should pay careful attention and provide appropriate interventions for the subdomains of HRQOL. In turn, this could help promote the health of older adults in South Korea.

The findings from our structural equation model clarified that depression, community resilience, social support, and disaster preparedness were associated with HRQOL among earthquake-affected older adults. Further, the model fit indices of the modified model were acceptable, confirming that the proposed conceptual model was supported. Depression, community resilience, and social support had significant direct and total effects on HRQOL. Depression and disaster preparedness had significant indirect and total effects on HRQOL. In contrast, PTSS had non-significant effects on HRQOL.

Depression showed significant negative direct, indirect, and total effects on HRQOL, which is consistent with the results of previous studies on older adults [9] and adults between 18 to 65 years [7, 22, 42]. Older adults with depression have difficulties adjusting to chaotic disaster situations and building social relationships with others in unfamiliar environments, such as temporary residential facilities [42]. The impact of depression might lead to reduced outdoor activities, job performance and increased use of healthcare services, which might subsequently result in lower HRQOL [42, 43]. The application of exercise programs developed for older adults who were affected by the 2011 earthquake and tsunami in Japan improved their mental health, which positively contributed to improving their HRQOL [43]. The key factors of the exercise program included being conducted by trained nurses and the promotion of exercise and social interactions. When applying the program for older adults, it may prove effective in strengthening their mental health by encouraging interest, social relationships, and physical activities [43].

Community resilience had significant positive direct and total effects on older adults’ HRQOL, which coincides with studies reporting that government support and policies [16], disaster preventive policies [18], and understanding of policy issues [44] are associated with increased HRQOL. Furthermore, community resilience mediated the effects of depression and disaster preparedness on HRQOL. This might be because community resilience stems from strong positive emotional reactions that can help an individual overcome negative emotions such as depression in disaster situations. In this way, community resilience might have served as a buffer for negative emotions owing to reduced HRQOL [15, 22]. Additionally, it is reported that people who were involved in education or volunteer to reduce disaster risk may have a higher awareness of community resilience [15]. These findings suggest that the application of community resilience improvement programs for vulnerable older adults might be an effective way to improve HRQOL and should be implemented by people with educational or volunteering experience in disaster risk management programs. Maintaining a supportive social network of community resources and educational training programs about disasters, risk prevention, risk reduction techniques, evacuation, and survival skills are valuable practices [15, 45].

Social support had significant positive direct and total effects on older adults’ HRQOL, which is aligned with previous studies’ findings that focused on the older adult group [9, 22] and adults in general [21, 22, 42]. Additionally, social support mediated the effects of depression on HRQOL, which is also consistent with previous research [22]. When depression negatively affects HRQOL, social support may have a buffering effect [21, 46]. As older people experience a sense of social isolation and social network disruption owing to the long-term effects of earthquakes [12], it is crucial to note that social support mitigates these negative effects on people’s HRQOL, thus alleviating both physical and psychological damage and resulting in increased HRQOL [21]. Social support provides practical assistance, such as short-term emergency relief in the immediate aftermath of earthquakes, as well as long-term healthcare and social services to reduce health deterioration and relieve stress [9, 21]. The results also revealed that social support scores decreased as time passed (from 3 months after the earthquake to about 1 year after the earthquake). These results suggest that social support may be high soon after a disaster but decreases over time. Therefore, strengthening the mid- to long-term social support system for vulnerable older adults may be an effective strategy to improve their HRQOL.

Disaster preparedness had significant positive indirect and total effects on HRQOL by mediating the effects of community resilience and social support. This suggests that disaster preparedness as part of disaster risk reduction activities in communities could indirectly result in increased HRQOL [15]. These results are consistent with a previous finding that disaster preparedness has positive associations with spiritual well-being, emotional well-being, and life satisfaction [19]. Moreover, disaster preparedness was associated with increased community resilience and social support, which implies that preventive measures and activities could strengthen the coping capacity of communities that are recovering from earthquake damage [15]. In light of these findings, the government and private institutions should continue to strengthen older adults’ disaster preparedness.

Finally, PTSS was not significantly associated with older adults’ HRQOL, which was inconsistent with previous findings [3, 42, 47–51]. The reason for this inconsistency might be that the magnitude of the Pohang earthquake was relatively small compared to those of earthquakes in previous studies, such as Van in Turkey or Sichuan in China [3, 51]. Consequently, the degree of damage to human life and well-being would have been relatively minor. However, PTSS is an important health issue for vulnerable older adults, and social interests are necessary to consider when screening high-risk groups and providing intervention programs at the community level [4]. The current study adds to the existing knowledge that it might be an effective strategy to provide disaster-relief psychological support programs that prioritize depression more than PTSS in cases when the earthquake causes relatively minor damage.

### Limitations

The findings should be understood within the context of the study limitations. First, the self-report responses from the older adults were obtained 1 year after the earthquake. Thus, it is difficult to rule out recall bias and memory deterioration. Second, this study is limited in its ability to identify the level of depression and associated factors before the earthquake. The results of the current study should be interpreted carefully. Third, the study’s cross-sectional design is a weakness because it cannot explain causal inferences. Longitudinal studies can provide more precise information on causal pathways. Fourth, although participants were selected through proportional quota sampling, data collection was conducted in places that were relatively easy to access, such as senior citizen centers, senior citizen schools, and temporary housing facilities. Additionally, some people whose houses were severely damaged were living elsewhere at the time the research was conducted. Thus, it is difficult to rule out the possibility that the results might have been overestimated or underestimated. Careful attention is required in generalizing the results to the entire older population of South Korea. Despite these limitations, the findings provide valuable evidence for current disaster implementation and recovery programs at disaster-relief psychological support centers and community healthcare centers.

## Conclusions

This study verified the path between associated factors and HRQOL among older adults who experienced the Pohang earthquake in South Korea by constructing a hypothetical model based on the HRQOL model developed by Wilson and Cleary [23] and revised by Zubritsky et al. [24]. The results of the modified model of the current study showed that lower depression and higher community resilience, social support, and disaster preparedness have significant effects on increased HRQOL. New perspectives on multidimensional HRQOL factors would be needed for a more comprehensive understanding on how HRQOL can be increased after an earthquake. When providing interventions to improve HRQOL among older adults, the focus should be on reducing depression and strengthening community resilience, social support, and disaster preparedness. These findings confirm existing disaster-related knowledge and provide a foundation for further research. Furthermore, they can be used as evidence to develop HRQOL improvement programs for older adults in community health centers and disaster-relief psychological support centers.

## Data Availability

All data generated or analyzed during this study are included in this manuscript.

## Abbreviations

ADL: Activities of daily living
CART: Communities Advancing Resilience Toolkit
CFI: Comparative fit index
GDS-15: Short form of the Geriatric Depression Scale-15
HRQOL: Health-related quality of life
IADL: Instrumental activities of daily living
PTSS: Posttraumatic stress symptoms
RMSEA: Root mean squared of approximation
SEM: Structural equation modeling
SRMR: Standard root mean residual
TLI: Tucker-Lewis index
WHOQOL-BREF: Abbreviated World Health Organization Quality of Life questionnaire
